# Prediction of Prolonged Length of Hospital Stay in Patients with Acute Exacerbations of Chronic Obstructive Pulmonary Disease: An Interpretable Machine Learning Tool

**DOI:** 10.3390/jcm15156127

**Published:** 2026-08-06

**Authors:** Jingjing Xiong, Lian Liu, Zhenni Chen, Weiling Cai, Jianjun Luo, Suli Liu, Jiangyue Qin, Fangying Chen, Xiaohua Li, Zhixin Qiu, Yongchun Shen

**Affiliations:** 1 Department of Pulmonary and Critical Care Medicine, West China Hospital, Sichuan University, Chengdu 610041, China; 2 State Key Laboratory of Respiratory Health and Multimorbidity, West China Hospital, Sichuan University, Chengdu 610041, China; 3 Division of Pulmonary Diseases, State Key Laboratory of Biotherapy of China, West China Hospital, Sichuan University, Chengdu 610041, China; 4 Department of Medical Administration, West China Hospital, Sichuan University, Chengdu 610041, China; 5 Department of Respiratory and Critical Care Medicine, Luojiang District People’s Hospital, Deyang 618000, China; 6 Department of Intensive Care Unit, People’s Hospital of Leshan, Leshan 614000, China; 7 Department of General Practice, West China Hospital, Sichuan University, Chengdu 610041, China; 8 Department of Tuberculosis, The Third People’s Hospital of Tibet Autonomous Region, Lhasa 850000, China; 9 Department of Respiratory and Critical Care Medicine, The Sixth People’s Hospital of Chengdu, Chengdu 610041, China

**Keywords:** acute exacerbation of chronic obstructive pulmonary disease, length of hospital stay, clinical prediction model, machine learning, SHapley Additive exPlanations

## Abstract

**Objective:** Reducing the length of hospital stay (LOS) is a core objective in the management of acute exacerbations of chronic obstructive pulmonary disease (AECOPD). This study aimed to develop an interpretable and clinically applicable machine learning tool for predicting prolonged LOS in this population. **Methods:** This retrospective three-center study enrolled 1342 patients, who were randomly allocated to a training set (70%) and a test set (30%). Candidate predictors were screened using least absolute shrinkage and selection operator (LASSO) regression, and six machine learning models were developed and compared: logistic regression, random forest, gradient boosting machine, CatBoost, support vector machine, and neural network. Model discrimination was evaluated using the area under the receiver operating characteristic curve (AUC), calibration plots, and decision curve analysis. Model interpretability was achieved through SHapley Additive exPlanations (SHAP) and subgroup analyses. **Results:** Prolonged LOS, defined as >10 days (the median LOS in the training set), occurred in 42.0% of patients. Nine predictors were identified, including daily inhaled medication use, sputum microbiological examination, antibiotic administration, corticosteroid therapy, diuretic use, ICU admission, oxygenation index, platelet count, and neutrophil count. The random forest model demonstrated superior and consistent discriminative performance, achieving an AUC of 0.778 (95% CI: 0.748–0.807) in the training set and 0.721 (95% CI: 0.672–0.771) in the test set. SHAP analysis ranked diuretic use, daily inhaled medication use, corticosteroid therapy, sputum microbiological examination, and antibiotic administration as the five most influential features, revealing treatment-related variables associated with prolonged LOS. Subgroup analyses indicated better predictive performance in lower-risk patients (age ≤ 65 years with eGFR 1–2 stage). **Conclusions:** Random Forest may enable to promptly identify prolonged LOS high-risk patients, enhancing clinical vigilance and optimizing healthcare resource allocation in AECOPD patients.

## 1. Introduction

Acute exacerbation is a critical event in chronic obstructive pulmonary disease (COPD), with significant morbidity, mortality, and healthcare resource consumption [1]. COPD is the third leading cause of death globally, and acute exacerbation of COPD (AECOPD) not only causes a sharp decline in patients’ quality of life but also serves as a major contributor to hospitalization, increasing medical costs, and poor prognosis [2,3]. A study indicated that patients with COPD experience 0.5~3.5 acute exacerbations annually [4]. Globally, 20~70% of AECOPD patients require hospital admission, and approximately half die within five years [5]. The 2026 Global Initiative for Chronic Obstructive Pulmonary Disease (GOLD) explicitly identifies reducing hospital stays and preventing readmissions as core objectives in the management of AECOPD [1]. Thus, predicting the length of hospital stay (LOS) for is crucial to improve clinical decisions and reduce healthcare costs.

Previous studies have explored potential determinants of LOS in AECOPD patients. Weekend admission and lack of respiratory care were linked to prolonged COPD stays [6]. A mMRC ≥ 2 and respiratory acidosis increased the risk of prolonged hospital stay [7]. Lower FEV_1_, a lack of home oxygen, heart failure, and an antibiotic delay of 2.3~3.1 days [8]. A 5-factor model in which high-risk patients, including those aged ≥ 75 years, mMRC ≥ 3, PaCO_2_ ≥ 55 mmHg, WBC > 10 × 10^9^/L, and heart failure, had an average LOS of 10.2 days [9]. These investigations consistently identify impaired lung function, cardiovascular comorbidities, and frequent exacerbations as critical factors of prolonged LOS. However, conventional regression lacks precision and generalizability in complex clinical data, and traditional clinical judgment is often similarly imprecise. This necessitates the development of robust, data-driven models that leverage multi-modal clinical and biomarker data to increase prognostic accuracy.

As a core branch of artificial intelligence, machine learning enables computers to automatically extract patterns and make predictions from data through algorithms, making it useful in medical image recognition, risk prediction, and treatment optimization [10,11,12]. Machine learning identifies complex patterns in data, improves predictions, and advances clinical decision-making [13,14]. However, machine learning models lack interpretability. To interpret the “black-box” model, SHapley Additive exPlanations (SHAP) leverages game theory to quantify each feature’s marginal contribution, detecting nonlinear relationships and potential interactions automatically to visualize decisions [15].

This study aims to develop and validate an interpretable machine learning model for predicting prolonged LOS in AECOPD patients by synthesizing readily available medical information including demographic characteristics, clinical variables, and biomarkers; identifying critical predictors of prolonged LOS; and providing a clinically actionable tool via SHAP and subgroup analyses.

## 2. Materials and Methods

### 2.1. Study Population

This retrospective study analyzed demographic and clinical data from patients discharged with a primary diagnosis of AECOPD between July 2019 and February 2024 at the Department of Pulmonary and Critical Care Medicine of three hospitals in Southwest China: West China Hospital, Luojiang District People’s Hospital, and People’s Hospital of Leshan. Patients were identified via the International Statistical Classification of Diseases and Related Health Problems, 10th Revision codes for AECOPD (ICD-10-J44.1) from the hospital’s electronic medical records system [16]. We followed the Transparent Reporting of a Multi-variable Prediction Model for Individual Prognosis or Diagnosis (TRIPOD+AI) reporting guideline and the Strengthening the Reporting of Observational Studies in Epidemiology (STROBE) statement (Supplementary Files S1 and S2). This study was approved by the Biomedical Research Ethics Committee of West China Hospital, Sichuan University, and written informed consent was waived because of its retrospective nature [approval date: 28 October 2025; approval number: 2025 (2201)].

### 2.2. Inclusion and Exclusion Criteria

With the code ICD-10-J44.1, the medical records were reviewed. The inclusion criteria were as follows: (1) Primary diagnosis of AECOPD based on the GOLD 2022 for the Diagnosis and Treatment of Chronic Obstructive Pulmonary Disease, in which AECOPD was defined as the acute worsening of respiratory symptoms in COPD patients, including increased dyspnea, accompanied by chest tightness, wheezing, aggravated cough, increased sputum volume, or changes in sputum color/viscosity, fever or other systemic symptoms [17]. (2) Age ≥ 40 years. (3) Hospitalized for 24 or more hours. (4) Complete baseline and clinical data. (5) As patients had multiple admissions, only data from the first hospitalization were collected. The exclusion criteria were as follows: (1) Patients whose respiratory difficulties were caused primarily by factors other than AECOPD, such as asthma, pneumothorax, pleural effusion, acute coronary syndrome, or pulmonary embolism. (2) Patients who died during hospitalization or were discharged against medical advice. (3) Patients’ baseline or clinical data were incomplete. (4) Patients’ extreme LOS (≥30 days).

### 2.3. Variables

The demographic data collected included sex, age, marital status, family history of COPD, smoking history, and daily inhaled medication use. The clinical data of the enrolled patients included vital signs (heart rate, respiratory rate, diastolic blood pressure, and systolic blood pressure), arterial blood gas analysis (potential of hydrogen, oxygenation index, arterial carbon dioxide tension, and bicarbonate ion), complete blood tests (hemoglobin concentration, platelet count, white blood cell count, neutrophil count, lymphocyte count, monocyte count, eosinophil count, and basophil count), liver function tests (total bilirubin, alanine aminotransferase, aspartate aminotransferase, alkaline phosphatase, total protein, and albumin), renal function tests (blood urea nitrogen, serum creatinine, and uric acid), serum electrolytes (calcium, sodium, potassium, chloride, and glucose), estimated glomerular filtration rateand (eGFR), N-terminal pro-B-type natriuretic peptide (NT-proBNP), hospital treatment details (sputum microbiological examination, antibiotic administration, corticosteroid therapy, diuretic use, noninvasive ventilation, and intensive care unit (ICU) admission), and LOS. Retrospectively, demographic data were collected from admission records and LOS from discharge records. Clinical data were defined as the first emergency department or first post-admission blood test results and hospital treatments from order lists.

eGFR was calculated using the CKD-EPI 2021 creatinine equation without race coefficient (Appendix A.1) and categorized into CKD stages (G1 = eGFR ≥ 90 mL/min/1.73 m^2^, G2 = eGFR 60–89 mL/min/1.73 m^2^, G3a = eGFR 45–59 mL/min/1.73 m^2^, G3b = eGFR 30–44 mL/min/1.73 m^2^, G4 = eGFR 15–29 mL/min/1.73 m^2^, G5 = eGFR < 15 mL/min/1.73 m^2^) based on standard KDIGO guidelines, then which were marked as numerical values (G1–G2 = 0, G3–G5 = 1) for statistical analysis [18,19,20]. Using the median LOS in the training set, outcome was defined as prolonged LOS greater than the median [6,21]. All variables were verified by three independent research assistants with agreement.

### 2.4. Statistical Analysis

#### 2.4.1. Baseline Analysis

All analyses were performed with R 4.5.0. The Shapiro–Wilk test was used to evaluate the normality of continuous variables. Normally distributed continuous variables are expressed as the means ± standard deviations, whereas nonnormally distributed continuous variables are expressed as medians [25th percentiles, 75th percentiles]. Continuous variables and outliers are presented by violin plots, but not manage outliers in order to ensure data authenticity. Categorical variables are presented as counts (percentages). All data were divided into a training set and test set at a 7:3 ratio, the former to develop models and the latter to validate. Distribution comparisons between the two sets were performed via the Kolmogorov–Smirnov test (*p* > 0.05) for continuous variables and the Chi–square test (*p* > 0.05) for categorical variables. The class imbalance problem was addressed using the Synthetic Minority Over-sampling Technique.

#### 2.4.2. Variable Selection

The meaningful variables selected by least absolute shrinkage and selection operator (LASSO) regression in the training set were retained for model development after confirming the absence of multicollinearity (variance inflation factor values < 3).

#### 2.4.3. Model Development, Assessment and Explanation

These features were used to develop six binary classification machine learning models (control LOS = 0, prolonged LOS = 1): logistic regression (Logistic), random forest (RF), gradient boosting machine (GBM), CatBoost, support vector machine (SVM), and neural network (NNet). Performance metrics including the area under the ROC curve (AUC), calibration plots, and decision curve analysis (DCA) were used to evaluate each model in both sets. After comprehensively comparing the models, the final model was selected, and SHAP values were used to visualize the feature importance and directionality of effects.

#### 2.4.4. Subgroup Analysis and Sensitivity Analyses

After explaining the final model with SHAP, subgroup analyses were conducted on the model, including gender and age (≤65 years, >65 years). Based on clinical significance and the most significant predictive variable identified by SHAP analysis, after independent decision-making by two senior experts, two variables were selected for subgroup analysis. To strengthen the model, an alternative threshold of 7 days was used in sensitivity analyses [22].

## 3. Results

### 3.1. Baseline Characteristics

**Figure 1:**
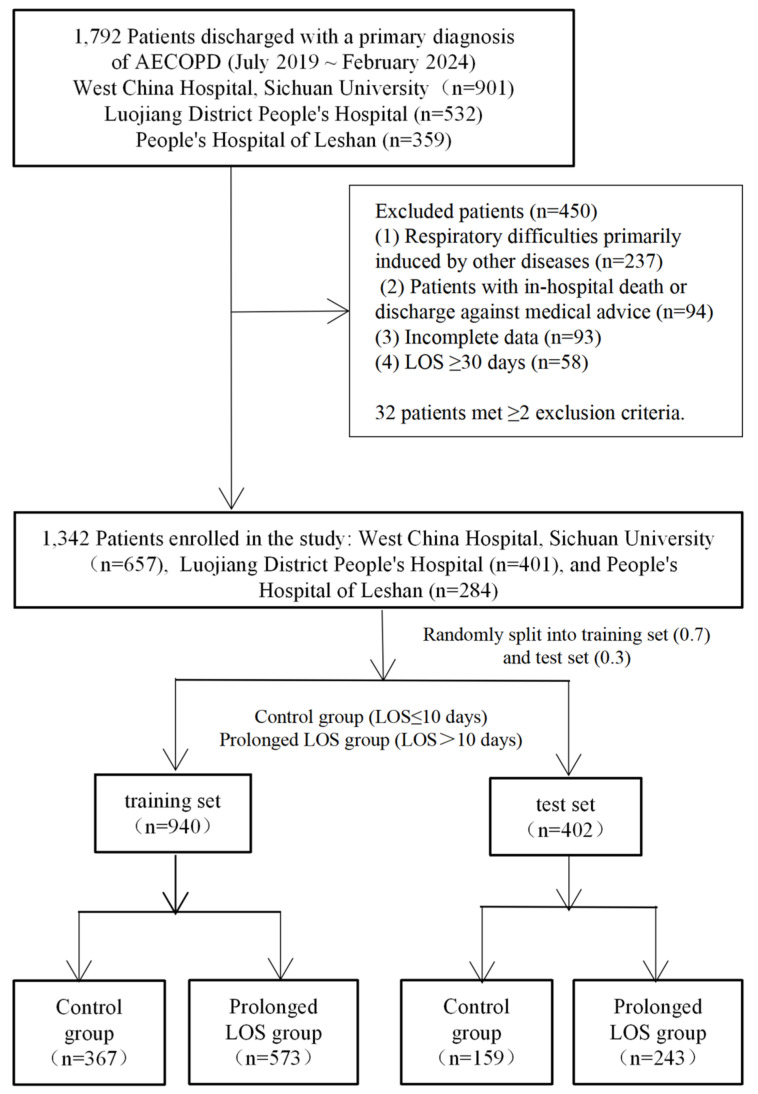
Flowchart of patients’ selection. LOS: length of hospital stay.

1792 records were retrieved from three databases; after screening the inclusion and exclusion criteria, 1342 patients, Asian (Chinese), were ultimately enrolled (Figure 1). In the overall dataset, the enrolled patients had a mean age of 73.55 ± 10.00 years, were predominantly male (68.2%), and were largely married (89.3%), with the vast majority being former or current smokers (91.3%). Therapeutically, most patients received antibiotic therapy (92.8%) and corticosteroid therapy (87.1%), while diuretics were used in 68.4% of patients. In addition, 20.9% exhibited positive sputum microbiological results, 19.2% required noninvasive ventilation, and 4.2% were admitted to the ICU. Median LOS was 9 days for the total cohort and the test set, whereas a slightly longer median of 10 days was observed in the training set. Distributional comparisons between the training and test sets revealed no significant differences across all covariates (all *p* > 0.05), confirming that the two subsets were balanced and suitable for model development and validation. All baseline characteristics are summarized in Table 1 and Supplemental Table S1. Continuous variables’ histograms were shown in Supplemental Figure S1.

### 3.2. Selection of Model Features

Patients were divided into a control group (LOS ≤ 10 days) and a prolonged LOS group (LOS > 10 days). In the training set, the prolonged LOS group comprised 73.1% males, 93.8% married individuals, and 88.4% with a smoking history, whereas 83.7% did not use daily inhaled medication. Their treatments featured high rates of antibiotic administration, corticosteroid therapy, and diuretic use, at 98.8%, 93.6%, and 85.2%, respectively. Clinically, these patients presented with a lower oxygenation index and higher rates of noninvasive ventilation use (25.9%) and ICU admission (7.2%). Detailed baseline characteristics of the two groups in the training set are presented in Supplemental Table S2.

**Figure 2:**
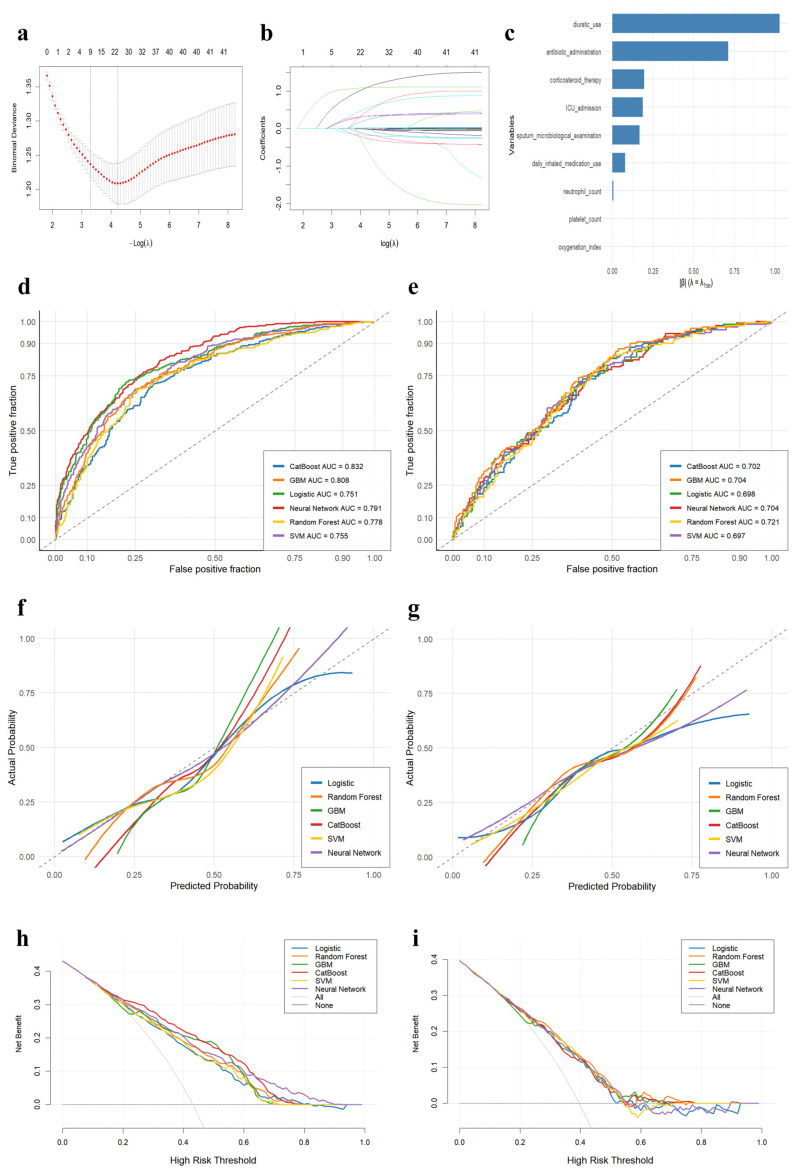
LASSO for variable selection and ROC, Calibration plots, and DCA of six machine learning models. (**a**) Cross-validation results of LASSO regression. (**b**) LASSO regression coefficient profile plot for log(λ). (**c**) Relative importance of variables by using LASSO regression. (**d**) ROC of six machine learning models for the control group and prolonged LOS group on the training set. (**e**) ROC of six machine learning models for the control group and prolonged LOS group on the test set. (**f**) Calibration plot of six machine learning models for the control group and prolonged LOS group on the training set. (**g**) Calibration plot of six machine learning models for the control group and prolonged LOS group on the test set. (**h**) DCA of six machine learning models for the control group and prolonged LOS group on the training set. (**i**) DCA of six machine learning models for the control group and prolonged LOS group on the test set. LASSO: Least absolute shrinkage and selection operator; ROC: Receiver operating characteristic curves; DCA: Decision curve analysis; LOS: length of hospital stay; Logistic: Logistic Regression; RF: Random Forest; GBM: Gradient Boosting Machine; SVM: Support Vector Machine; NNet: Neural Network.

As shown in Figure 2a–c, via LASSO and multicollinearity assessment, nine effective variables, including diuretic use, antibiotic administration, corticosteroid therapy, ICU admission, sputum microbiological examination, daily inhaled medication use, neutrophil count, platelet count, and oxygenation index, were selected to develop and validate models.

### 3.3. Evaluation and Comparison of the Six Models

For logistic, RF, GBM, CatBoost, SVM and NNet, Figure 2d depicts the ROC curves of the training set, while Figure 2e shows the ROC curves for the test set. Similarly, calibration plots (Figure 2f,g) and DCA curves (Figure 2h,i) illustrate the performance of various models. In the training set, CatBoost demonstrated the highest AUC at 0.832 (95% CI: 0.806–0.854), followed by GBM (0.808, 95% CI: 0.780–0.836), NNet (0.791, 95% CI: 0.762–0.819), RF (0.778, 95% CI: 0.748–0.807), SVM (0.755, 95% CI: 0.724–0.786) and Logistic (0.751, 95% CI: 0.720–0.782). When evaluated on the test set, the performance deteriorated relative to their training-set estimates. The AUCs for CatBoost, GBM, NNet, RF, SVM, and Logistic were 0.702 (95% CI: 0.647–0.754), 0.704 (95% CI: 0.654–0.755), 0.704 (95% CI: 0.653–0.755), 0.721 (95% CI: 0.672–0.771), 0.697 (95% CI: 0.646–0.748), and 0.698 (95% CI: 0.647–0.749), respectively. Table 2 presents comprehensive classification performance metrics. RF (Appendix A.2) was selected as the final model based on its better balance between discriminative performance and generalisability among six models.

### 3.4. Explanation of the RF Model on the Test Set

#### 3.4.1. Visualization of Feature Importance

**Figure 3:**
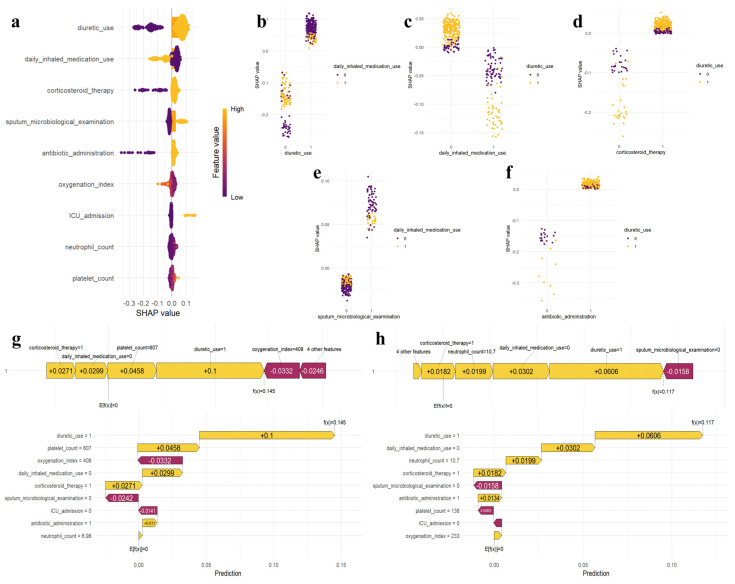
SHAP Summary Plot, interaction effects of the top 5 features, SHAP force plot, and the SHAP waterfall plot. (**a**) SHAP summary plot. (**b**) Interaction effects of diuretic use. (**c**) Interaction effects of daily inhaled medication use. (**d**) Interaction effects of corticosteroid therapy. (**e**) Interaction effects of sputum microbiological examination. (**f**) Interaction effects of antibiotic administration. (**g**) SHAP force plot and SHAP waterfall plot of the 1st patient. (**h**) SHAP force plot and SHAP waterfall plot of the 100th patient. SHAP: SHapley Additive exPlanations; LOS: length of hospital stay.

SHAP analyzed the contributions of the nine features to the prediction; the rankings were then quantified by SHAP values in descending order (Figure 3a), emphasizing the top five features: diuretic use, daily inhaled medication use, corticosteroid therapy, sputum microbiological examination, and antibiotic administration. The importance of the other variables subsequently decreased in that order. SHAP value plots of each variable are put in Supplemental Figure S2.

#### 3.4.2. Interaction Effects of the Top 5 Features

The interaction effects of these 5 features had significant contributions to the prediction. Figure 3b–f illustrates the relationships among these features. The predictive effect of diuretic use is context-dependent. It was connected with the low risk of prolonged LOS among patients without diuretic use.

#### 3.4.3. Decision-Making Process of Individual Prediction

The SHAP force plot and waterfall plot are used to determine how each clinical feature contributes to the final prediction, revealing the decision-making pathway. Figure 3g,h shows the contribution of each feature to the model prediction for the 1st and 10th patients. Each feature and corresponding SHAP value indicates the positive or negative impact on the prediction result. In Figure 3g, diuretic use, higher platelet count, antibiotic administration, and corticosteroid therapy made significant contributions to the prolonged LOS, whereas the oxygenation index contributed to the control LOS. Six other features also had varying degrees of impact.

### 3.5. Subgroup Analysis of the RF Model on the Test Set

**Figure 4:**
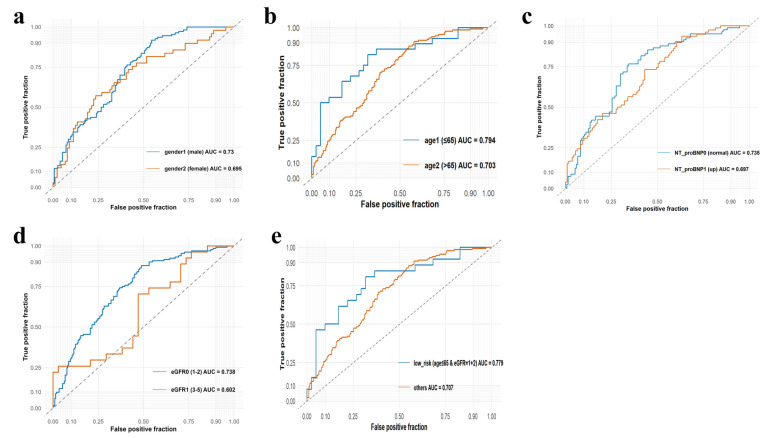
Subgroup analysis on the test set and ROCs. (**a**) Sub-gender (female, male) analysis. (**b**) Sub-age (≤65 years, >65 years) analysis. (**c**) Sub-NT-proBNP (below the maximum detection value, above the maximum detection value) analysis. (**d**) Sub-eGFR (≥60 mL/min/1.73 m^2^, ≤59 mL/min/1.73 m^2^) analysis. (**e**) Sub-low-risk (age ≤ 65 years and eGFR0) analysis. ROC: receiver operating characteristic curves. NT-proBNP: N-terminal pro-B-type natriuretic peptide. eGFR: estimated glomerular filtration rateand, calculated using the CKD-EPI 2021 creatinine equation; marked as numerical value (≥90 mL/min/1.73 m^2^ = 1, 60–89 mL/min/1.73 m^2^ = 2, ≤59 mL/min/1.73 m^2^ = 3) for statistical analysis.

Subgroup analysis based on gender (male, female) revealed that the predictive performance of RF was better (Figure 4a). For patients ≤ 65 years old, the AUC reached 0.794, compared to 0.703 for patients > 65 years old (Figure 4b). Based on the highlighted variable, diuretic use, two experts recommended incorporating NT-proBNP and eGFR for subgroup analyses. RF demonstrated higher predictive performance for patients with normal NT-proBNP (AUC: 0.730) and normal eGFR (AUC: 0.738) (Figure 4c,d). Building on the single-subgroup analysis, a comprehensive subgroup incorporating age and eGFR revealed that RF had stronger predictive performance (AUC: 0.779) in the low-risk group (≤65 years old and normal eGFR) compared to other groups (AUC: 0.707) (Figure 4e). Subgroups were shown in Table 2 and Supplementary Figure S3.

### 3.6. Sensitivity Analyses of the RF Model

Sensitivity analyses using a LOS cut-off of 7 days were conducted to assess result robustness, with all outputs summarized in Supplementary Figure S4 and Supplementary Table S3. The LOS > 7 days model had a test-set AUC of 0.731 (95% CI 0.673–0.788), versus 0.721 (95% CI 0.672–0.771) for the primary LOS > 10 days model. The two thresholds yielded nearly identical key predictive results and consistent association trends across subgroups, with no reversed risk effects. These findings confirm relatively stable prediction patterns whether using a fixed 7-day cut-off or the training-set-derived 10-day threshold.

## 4. Discussion

This multicentre retrospective study was to develop and interpret a machine learning tool for predicting prolonged LOS in patients with AECOPD. LASSO identified 9 meaningful predictors: diuretic use, antibiotic administration, corticosteroid therapy, ICU admission, sputum microbiological examination, daily inhaled medication use, neutrophil count, platelet count, and oxygenation index. The RF model was better than the other five models, as evidenced by the smaller decline in AUC from training to test (Δ = 0.057) and achieved the highest test-set AUC. CatBoost, despite achieving the highest training-set AUC (0.832), exhibited a substantially large attenuation upon external validation (Δ = 0.130). Accordingly, the RF was deemed to represent the balanced compromise between discriminative accuracy and transportability. SHAP interpreted and suggested diuretic use, daily inhaled medication use, corticosteroid therapy, sputum microbiological examination, and antibiotic administration as the top five important features, closely associated with treatments, solving the lack of interpretability in RF [23,24]. To further explore potential heterogeneity in the predictive contributions of features, we examined SHAP dependence plots, which revealed a trend towards simpler treatments being associated with control LOS. These factors likely correlate with disease severity: more critical conditions and complex treatments are associated with higher risks, which is consistent with treatments being used as indicators of disease severity [25]. Recent studies have demonstrated that transparent, interpretable machine learning models can outperform conventional scoring systems in predicting dynamic clinical trajectories [26].

Notably, the top five SHAP-identified factors reflect in-hospital therapeutic decisions rather than information available at the point of admission. We readily acknowledge that the model is not designed as a triage tool. However, we contend that this does not diminish its clinical utility. Rather, these variables serve as dynamic surrogate markers of evolving disease severity, because clinical decisions to initiate diuretics, antibiotics, or corticosteroids are evidence-based professional judgments informed by the patient’s deteriorating or improving physiological trajectory. Therefore, the model’s value should be framed as an early in-hospital dynamic risk-reassessment tool, applicable after the initial professional evaluation when the therapeutic trajectory has been established. During this window, the model can provide quantitative support for bed allocation, early rehabilitation referral, and discharge planning. These decisions remain clinically actionable beyond the point of admission. This framing aligns with the emerging paradigm that predictive tools in care need not be restricted to admission triage, but can augment clinical decision-making throughout the hospital course [27,28,29].

Pathophysiologically, these nine factors can be stratified into actionable therapeutic nodes, non-modifiable risk markers, and ICU admission, which moves beyond the observation that “sicker patients stay longer.” Among the actionable variables (diuretic use, daily inhaled medication use, corticosteroid therapy, sputum microbiological examination, and antibiotic administration), SHAP dependence plots revealed that the diuretic-related effect was pronounced among patients not receiving antibiotics or corticosteroids, or already established on daily inhaled therapy. A clinical nuance was found that the contribution of any therapeutic agent cannot be interpreted in isolation but must be contextualized within the patient’s overall inflammatory and infectious burden. Daily inhaled maintenance therapy demonstrated a modest buffering effect, attenuating the association between sputum microbiology positivity or diuretic use and prolonged LOS. This observation is biologically plausible, as regular airway maintenance reduces chronic airway inflammation and mucus hypersecretion, potentially mitigating exacerbation severity and consequent need for intensive interventions [30,31,32]. It suggests a potential interaction between baseline airway maintenance and acute infection, but the hypothesis warrants prospective validation. The non-modifiable risk markers (neutrophil count, platelet count, and oxygenation index) reflect the inflammatory burden and gas-exchange impairment, constituting the patient’s intrinsic risk endowment. Neutrophilia and thrombocytosis are well-established systemic inflammatory responders in AECOPD, while a low oxygenation index directly quantifies the severity of ventilation-perfusion mismatch [33,34]. ICU admission, though partly determined by institutional practice, serves as both an ultimate marker of severity and a proxy for resource intensity. This stratified interpretation transforms the model from severity into a clinically actionable decision-support tool. It both tells clinicians which patients are sick and quantifies which factors, under which clinical contexts, carry the greatest weight in driving prolonged LOS risk, thereby informing targeted early interventions.

Subgroup analyses revealed that model performance was enhanced in a low-risk type (age ≤ 65 years with preserved renal function, eGFR stages 1–2), achieving an AUC of 0.779 compared with 0.707 in the other cohort for the 10-day model. This finding suggests that prediction models may achieve higher accuracy when applied to clinical subpopulations, and supports future efforts toward phenotype-specific model refinement. In a sensitivity analysis using a 7-day threshold, the 7-day model yielded broadly comparable AUCs, supporting the robustness of our findings to threshold selection. But the two thresholds exhibited different characteristics that the 7-day model prioritized sensitivity at the expense of specificity (95.7% vs. 22.3%), whereas the 10-day model offered a more balanced trade-off (69.8% vs. 63.0%). A 7-day cutoff would risk generating excessive false-positive alerts. We therefore retained the training-set median (10 days) as the primary definition, which provides a more pragmatic balance for real-world respiratory ward application. Although direct comparisons with previously published AECOPD prediction models are challenging due to substantial heterogeneity in study populations, outcome definitions, and data sources, our test-set AUC of 0.721 falls within the range of prior estimates [35,36]. Head-to-head comparisons within the same validation cohort are warranted to establish a benchmarking framework in the future.

Given Clinical implementation, the moderate test-set AUC (0.721, 95% CI: 0.672–0.771) and the wide overlap of 95% CI across models underscore that this model should be viewed as a supplementary risk-screening tool rather than a diagnostic test. The model’s primary clinical utility resides in detecting patients whose therapeutic escalation carries an elevated risk of prolonged LOS, enabling intensified monitoring, vigilant tracking of clinical trajectories, enhanced supportive care, earlier discharge planning, and more judicious resource allocation. The model does not aim to replace clinical judgment, but to augment it by providing quantitative, data-driven support for dynamic risk identification.

Several limitations should be acknowledged. Firstly, the >10-day cutoff for prolonged LOS was derived from the median LOS in the training set. We acknowledge the inherent circularity. However, no universally accepted standardized cutoff exists for AECOPD LOS. Previous studies have employed different thresholds depending on cohort-specific distributions [7]. In addition, a rigorous comparison cannot be performed in the current study. Previously published predictive models adopt heterogeneous populations, variable inclusion criteria, distinct LOS-definition thresholds, and diverse modeling pipelines. Most published models lack the complete and transparent parameter and cohort details required for standardized cross-study benchmarking, prohibiting rigorous performance comparison. Secondly, six models demonstrated AUC declines from training to testing, indicative of overfitting. This overfitting may be attributed primarily to the retrospective nature rather than to insufficient model regularization. Although the RF achieved a better balance between discrimination and generalisability, its overall performance remains modest, highlighting the need for further external validation. Thirdly, SHAP analysis cannot support causal inference. Interpretations of diuretics or daily inhaled therapy should be considered hypothesis-generating. Fourthly, several established social and functional covariates were absent from our analysis, including frailty status, formal social support networks, and home-based care resources. Marital circumstances and social support are recognized modifiers of discharge readiness and inpatient recovery trajectories in patients with COPD, while frailty independently exacerbates the risk of prolonged admission [37,38,39]. Furthermore, retrospective data collection is prone to incomplete documentation, which restricts granular interpretation of predictive effects. Fifthly, for patients with multiple comorbid acute conditions (e.g., concurrent AECOPD and acute decompensated heart failure), subtle inter-center differences in diagnostic prioritization and coding habits may lead to inconsistent primary diagnosis classification. Specifically, detailed clinical records of multiple key respiratory support modalities were not fully captured in our retrospective database. We could not systematically document invasive mechanical ventilation (IMV) for patients who fail NIV [40], nor did we have access to data on high-flow nasal cannula (HFNC) as an alternative or complementary strategy for patients intolerant of NIV or those without acidotic hypercapnic failure [41], or extracorporeal CO_2_ removal (ECCO_2_R) for patients who fail or are at high risk of failing NIV [42]. Furthermore, the absence of documented hypercapnia or respiratory acidosis at admission, a key criterion for NIV indication [43], precluded formal assessment of NIV appropriateness. This limits the comprehensiveness of our respiratory support evaluation and may have underestimated the impact of advanced respiratory interventions on LOS. We intend to collect complete respiratory support data in the future to address this deficiency and further improve the clinical applicability. Sixthly, the lack of external validation in an independent cohort is a limitation shared by many published machine learning-based prediction models [44,45]. In the future, it is necessary to incorporate geographically and temporally distinct cohorts.

## 5. Conclusions

This study has developed a transparent, interpretable machine learning tool for predicting prolonged LOS in AECOPD patients. It enables serial risk identification and reassessment throughout hospitalization, prompting closer monitoring, heightened clinical vigilance, and enhanced supportive care, thereby facilitating earlier discharge planning and resource optimization.

## Figures and Tables

**Table 1:** Baseline characteristics of all data.

Variables	Total (n = 1342)	Training Set (n = 940)	Test Set (n = 402)	*p* Value
gender				0.220
male = 1	915 (68.2%)	651 (69.3%)	264 (65.7%)	
female = 2	427 (31.8%)	289 (30.7%)	138 (34.3%)	
age (years)	73.55 ± 10.00	73.48 ± 9.89	73.72 ± 10.26	0.711
marital status				1
married = 1	1199 (89.3%)	840 (89.4%)	359 (89.3%)	
other (unmarried/divorced/widowed) = 2	143 (10.7%)	100 (10.6%)	43 (10.7%)	
family history of COPD				0.739
no = 0	1335 (99.5%)	936 (99.6%)	399 (99.3%)	
yes = 1	7 (0.5%)	4 (0.4%)	3 (0.7%)	
smoking history				0.759
non-smoker = 0	117 (8.7%)	80 (8.5%)	37 (9.2%)	
ex-smoker/current smoker = 1	1225 (91.3%)	860 (91.5%)	365 (90.8%)	
daily inhaled medication use				0.303
no = 0	982 (73.2%)	696 (74.0%)	286 (71.1%)	
yes = 1	360 (26.8%)	244 (26.0%)	116 (28.9%)	
heart rate (bmp/min)	91.39 ± 18.64	91.84 ± 18.18	90.31 ± 19.66	0.321
respiratory rate (bmp/min)	20 [20, 21]	20 [20, 21]	20 [20, 21]	0.996
diastolic blood pressure (mmHg)	77 [68, 85]	77 [68, 80]	77 [68, 85]	0.980
systolic blood pressure (mmHg)	128 [113, 142]	128 [113, 142]	126 [114, 141]	0.722
potential of Hydrogen	7.41 [7.38, 7.44]	7.41 [7.38, 7.44]	7.41 [7.38, 7.44]	0.929
oxygenation index	255.01 ± 94.77	255.37 ± 93.71	254.17 ± 97.32	0.841
arterial carbon dioxide tension (mmHg)	43.00 [37.00, 51.85]	42.80 [37.00, 52.00]	43.50 [37.00, 51.60]	0.587
bicarbonate ion (mmol/L)	28.23 ± 6.43	28.19 ± 6.54	28.30 ± 6.18	0.314
hemoglobin concentration (g/L)	134.00 [119.00, 147.00]	135.00 [119.00, 147.00]	134.00 [117.00, 147.25]	0.889
platelet count (10^9^/L)	171.00 [128.00, 222.00]	170.00 [129.00, 223.75]	172.00 [126.75, 219.00]	0.842
white blood cell count (10^9^/L)	7.59 [5.75, 9.89]	7.41 [5.71, 9.81]	7.75 [5.84, 10.10]	0.094
neutrophil count (10^9^/L)	5.64 [4.02, 8.02]	5.52 [3.99, 7.89]	5.93 [4.17, 8.39]	0.190
lymphocyte count (10^9^/L)	1.02 [0.66, 1.47]	1.03 [0.68, 1.48]	1.01 [0.63, 1.45]	0.113
monocyte count (10^9^/L)	0.49 [0.34, 0.68]	0.50 [0.34, 0.68]	0.49 [0.34, 0.68]	0.898
eosinophil count (10^9^/L)	0.06 [0.02, 0.15]	0.06 [0.02, 0.15]	0.06 [0.01, 0.15]	0.731
basophil count (10^9^/L)	0.02 [0.01, 0.03]	0.02 [0.01, 0.03]	0.02 [0.01, 0.03]	0.464
total bilirubin (umol/L)	9.70 [6.80, 14.40]	9.70 [6.80, 14.68]	9.70 [6.88, 14.10]	0.897
alanine aminotransferase (U/L)	19.00 [13.00, 29.72]	19.00 [13.00, 29.23]	19.00 [13.26, 30.00]	0.576
aspartate aminotransferase (U/L)	23.00 [18.00, 32.00]	23.00 [18.00, 31.75]	24.00 [18.75, 33.00]	0.444
alkaline phosphatase (U/L)	82.00 [65.00, 101.00]	81.00 [65.00, 99.63]	85.00 [65.86, 104.00]	0.225
total protein (g/L)	65.84 ± 7.78	65.89 ± 7.80	65.70 ± 7.76	0.873
albumin (g/L)	37.70 [34.00, 41.00]	37.70 [34.00, 40.80]	37.60 [34.08, 41.40]	0.858
blood urea nitrogen (mmol/L)	6.20 [4.70, 8.11]	6.19 [4.69, 8.00]	6.30 [4.80, 8.39]	0.429
serum creatinine (umol/L)	72.80 [60.00, 89.00]	72.80 [60.00, 88.98]	72.60 [61.00, 91.00]	0.565
uric acid (umol/L)	338.14 ± 139.98	338.44 ± 139.94	337.44 ± 140.24	0.919
calcium (mmol/L)	2.21 [2.12, 2.31]	2.21 [2.12, 2.31]	2.22 [2.11, 2.33]	0.535
sodium (mmol/L)	140.00 [137.00, 142.20]	140.00 [137.00, 142.18]	140.00 [137.00, 142.00]	0.929
potassium (mmol/L)	3.99 ± 0.56	3.98 ± 0.57	3.99 ± 0.52	0.516
chloride (mmol/L)	102.00 [98.30, 105.00]	102.00 [98.73, 105.00]	102.00 [97.70, 105.00]	0.468
glucose (mmol/L)	6.27 [5.05, 8.10]	6.20 [4.99, 8.13]	6.36 [5.13, 8.07]	0.375
NT-proBNP (pg/mL)				0.576
below the maximum detection value = 0	735 (51.77%)	520 (55.32%)	215 (53.48%)	
above the maximum detection value = 1	607 (45.23%)	420 (44.68%)	187 (46.52%)	
eGFR (mL/min/1.73 m^2^)				0.133
G1(≥90) = 1	740 (55.14%)	517 (55.00%)	223 (55.47%)	
G2(60–89) = 2	431 (32.12%)	313 (33.30%)	118 (29.35%)	
G3a-G5 (≤59) = 3	171 (12.74%)	110 (11.70%)	61 (15.18%)	
sputum microbiological examination				0.962
negative = 0	1061 (79.1%)	744 (79.1%)	317 (78.9%)	
positive = 1	281 (20.9%)	196 (20.9%)	85 (21.1%)	
antibiotic administration				0.898
no = 0	97 (7.2%)	69 (7.3%)	28 (7.0%)	
yes = 1	1245 (92.8%)	871 (92.7%)	374 (93.0%)	
corticosteroid therapy				0.555
no = 0	173 (12.9%)	125 (13.3%)	48 (11.9%)	
yes = 1	1169 (87.1%)	815 (86.7%)	354 (88.1%)	
diuretic use				0.223
no = 0	424 (31.6%)	307 (32.7%)	117 (29.1%)	
yes = 1	918 (68.4%)	633 (67.3%)	285 (70.9%)	
non-invasive ventilation application				0.906
no = 0	1084 (80.8%)	758 (80.6%)	326 (81.1%)	
yes = 1	258 (19.2%)	182 (19.4%)	76 (18.9%)	
ICU admission				0.900
no = 0	1285 (95.8%)	901 (95.9%)	384 (95.5%)	
yes = 1	57 (4.2%)	39 (4.1%)	18 (4.5%)	
LOS (days)	9 [7.00, 13.00]	10 [7, 14]	9 [7, 13]	0.909

Values are expressed as the mean ± standard deviation or the median [interquartile range]. LOS: length of hospital stay.

**Table 2:** Comprehensive classification performance metrics of six models.

Model	AUC	AUC_95%CI_L	AUC_95CI_H	Accuracy	Balanced Accuracy	Sensitivity (Recall)	Specificity	PPV	NPV	F1	MCC	Brier	Kappa
Logistic ^a^	0.751	0.72	0.782	0.701	0.702	0.706	0.697	0.638	0.758	0.671	0.4	0.202	0.398
Logistic ^b^	0.698	0.647	0.749	0.629	0.63	0.635	0.626	0.526	0.724	0.575	0.255	0.214	0.252
RF ^a^	0.778	0.748	0.807	0.709	0.715	0.76	0.669	0.635	0.787	0.692	0.426	0.194	0.42
RF ^b^	0.721	0.672	0.771	0.657	0.664	0.698	0.63	0.552	0.761	0.617	0.321	0.206	0.313
GBM ^a^	0.808	0.78	0.836	0.753	0.747	0.704	0.791	0.718	0.779	0.711	0.496	0.191	0.496
GBM ^b^	0.704	0.654	0.755	0.647	0.634	0.572	0.695	0.552	0.713	0.562	0.266	0.213	0.266
CatBoost ^a^	0.832	0.806	0.854	0.74	0.739	0.731	0.748	0.687	0.786	0.708	0.476	0.178	0.475
CatBoost ^b^	0.702	0.647	0.754	0.634	0.628	0.597	0.658	0.534	0.714	0.564	0.252	0.213	0.251
SVM ^a^	0.755	0.724	0.786	0.699	0.706	0.753	0.658	0.625	0.779	0.683	0.407	0.201	0.401
SVM ^b^	0.697	0.646	0.748	0.644	0.652	0.692	0.613	0.539	0.753	0.606	0.298	0.213	0.291
Nnet ^a^	0.791	0.762	0.819	0.719	0.715	0.689	0.742	0.669	0.759	0.679	0.43	0.185	0.429
Nnet ^b^	0.704	0.653	0.755	0.649	0.647	0.635	0.658	0.549	0.734	0.589	0.288	0.214	0.286

^a^: train set. ^b^: testing set. Logistic: Logistic Regression; RF: Random Forest; GBM: Gradient Boosting Machine; SVM: Support Vector Machine; NNet: Neural Network.

## Data Availability

No data are available. The analytical code is available upon reasonable request. However, the individual-level data cannot be shared due to data use agreement restrictions. Both browser and WeChat access the online prediction URL for two models: https://xjj-app.shinyapps.io/LOS10app/ (accessed on 22 July 2026) and https://xjj-app.shinyapps.io/LOS7app/ (accessed on 22 July 2026).

## Supplementary Materials

The following supporting information can be downloaded at: https://www.mdpi.com/article/10.3390/jcm15156127/s1, Supplemental Figure S1: Histogram of continuous variables; Supplemental Figure S2: SHAP value plots of every variable in Random Forest; Supplemental Figure S3: SHAP summary plot of subgroup analysis; Supplemental Figure S4: Sensitivity analyses (LOS7 vs. LOS10); Supplemental Table S1: Baseline characteristics of three hospitals; Supplemental Table S2: Baseline characteristics of training set; Supplemental Table S3: Sensitivity analyses (LOS7 vs. LOS10); Supplementary File S1: TRIPOD+AI checklist; Supplementary File S2: STROBE_checklist [46].

## Abbreviations

COPD, chronic obstructive pulmonary disease; AECOPD, acute exacerbation of COPD; GOLD, Global Initiative for Chronic Obstructive Pulmonary Disease; LOS, length of hospital stay; SHAP, SHapley Additive exPlanations; eGFR, estimated glomerular filtration rateand; NT-proBNP, N-terminal pro-B-type natriuretic peptide; ICU, intensive care unit; CKD, Epidemiology Collaboration; CKD-EPI, Chronic Kidney Disease Epidemiology Collaboration; KDIGO, Kidney Disease: Improving Global Outcomes; LASSO, least absolute shrinkage and selection operator; Logistic, logistic regression; RF, random forest; GBM, gradient boosting machine; SVM, support vector machine; NNet, neural network; AUC, area under the curve; DCA, decision curve analysis; CI, confidence interval; IMV, invasive mechanical ventilation; HFNC, high-flow nasal cannula; ECCO_2_R, extracorporeal CO_2_ removal.

## Appendix A. Mathematical Formulation of the Predictive Model

### Appendix A.1. Estimated Glomerular Filtration Rate (eGFR) via the Race-Free 2021 CKD-EPI Creatinine Equation

$$\mathrm{eGFR}=142\times \min{\left(\frac{\mathrm{SCr}}{\kappa },1\right)}^{\alpha }\times \max{\left(\frac{\mathrm{SCr}}{\kappa },1\right)}^{-1.200}\times 0.9938^{\mathrm{Age}}\times 1.012\;(\mathrm{femaleonly})$$

Here, SCr = standardized serum creatinine (mg/dL); κ = 0.7 for women, κ = 0.9 for men; α = −0.241 for women, α = −0.302 for men; Age = patient age in years; The multiplier 1.012 is applied exclusively to female individuals. All eGFR outputs were reported in mL/min/1.73 m^2^ and stratified into KDIGO CKD stages G1 to G5.

### Appendix A.2. Random Forest Algorithm

The RF employed 10-fold cross-validation (repeated 3 times) combined with grid search to optimize hyperparameters, with the AUC as the core evaluation metric. Hyperparameter search covered node-splitting feature count, splitting rule, and minimum node sample size, ultimately selecting the optimal parameter combination: mtry = 3, splitrule = extratrees, and min node size = 70. The model integrated 1500 independent decision trees, each constructed using 50% training samples via resampling with replacement (replace = TRUE). Additionally, a regularization factor of 0.3 was applied to mitigate overfitting, and feature importance was assessed through permutation testing (importance = “permutation”). Each tree was constructed using 50% of the training samples through resampling with replacement, and feature importance was assessed via permutation testing. The model integrates the prediction results of decision trees using a probability-averaging rule (since ranger with probability estimation outputs class probabilities, rather than simple majority voting), ultimately achieving binary classification prediction for LOS outcomes. The core integration formula is as follows:

$$H(x)=\arg\underset{y\in \left\{0,1\right\}}{\max}\;\frac{1}{1500}\sum_{k=1}^{1500}{\hat{p}}_{k}\left(y|x,\Theta_{k}\right)$$

Here, *x* denotes a 9-dimensional feature vector (as indicated by the 9 predictors in the model), *y* represents the outcome variable (0 = No, 1 = Yes for prolonged LOS), *p*^*k*(*y*∣*x*,Θ*k*) indicates the predicted probability of class *y* from the *k*-th decision tree, and the final risk level is determined by selecting the category with the highest average predicted probability across all decision trees.
