# Supplementary material for: Prediction of Prolonged Length of Hospital Stay in Patients with Acute Exacerbations of Chronic Obstructive Pulmonary Disease: An Interpretable Machine Learning Tool
Source: J Clin Med. 2026 Aug 6;15(15):6127. doi: 10.3390/jcm15156127 (PMC13467324; doi:10.3390/jcm15156127)
Supplement: Supplementary file 1 [file jcm-15-06127-s001.zip › Supplemental Figure S1 Histogram of continuous variables.pdf]

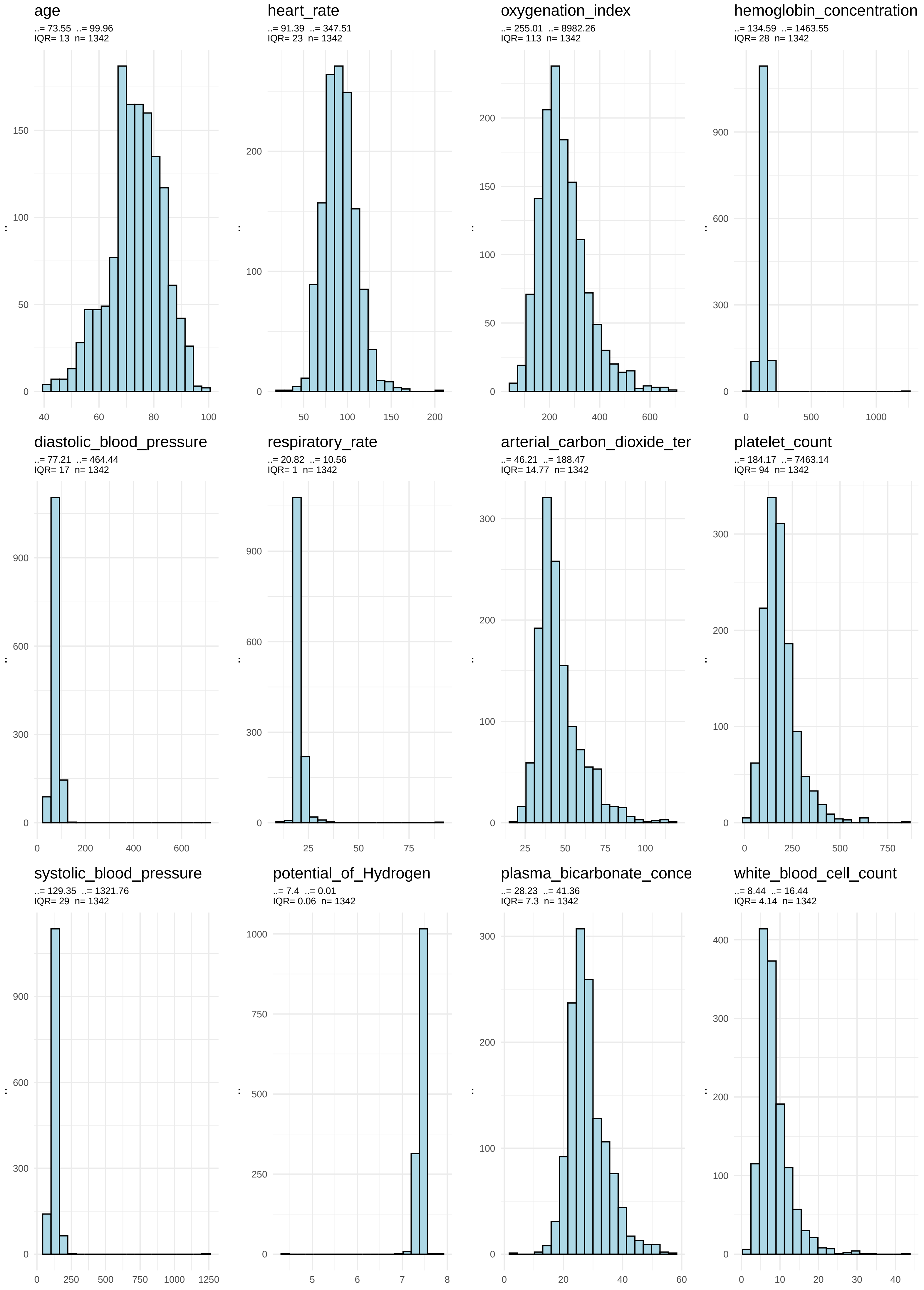
age
..= 73.55 ..= 99.96
IQR= 13 n= 1342
heart_rate
..= 91.39 ..= 347.51
IQR= 23 n= 1342
oxygenation_index
..= 255.01 ..= 8982.26
IQR= 113 n= 1342
hemoglobin_concentration
..= 134.59 ..= 1463.55
IQR= 28 n= 1342
diastolic_blood_pressure
..= 77.21 ..= 464.44
IQR= 17 n= 1342
respiratory_rate
..= 20.82 ..= 10.56
IQR= 1 n= 1342
arterial_carbon_dioxide_ter
..= 46.21 ..= 188.47
IQR= 14.77 n= 1342
platelet_count
..= 184.17 ..= 7463.14
IQR= 94 n= 1342
systolic_blood_pressure
..= 129.35 ..= 1321.76
IQR= 29 n= 1342
potential_of_Hydrogen
..= 7.4 ..= 0.01
IQR= 0.06 n= 1342
plasma_bicarbonate_conce
..= 28.23 ..= 41.36
IQR= 7.3 n= 1342
white_blood_cell_count
..= 8.44 ..= 16.44
IQR= 4.14 n= 1342

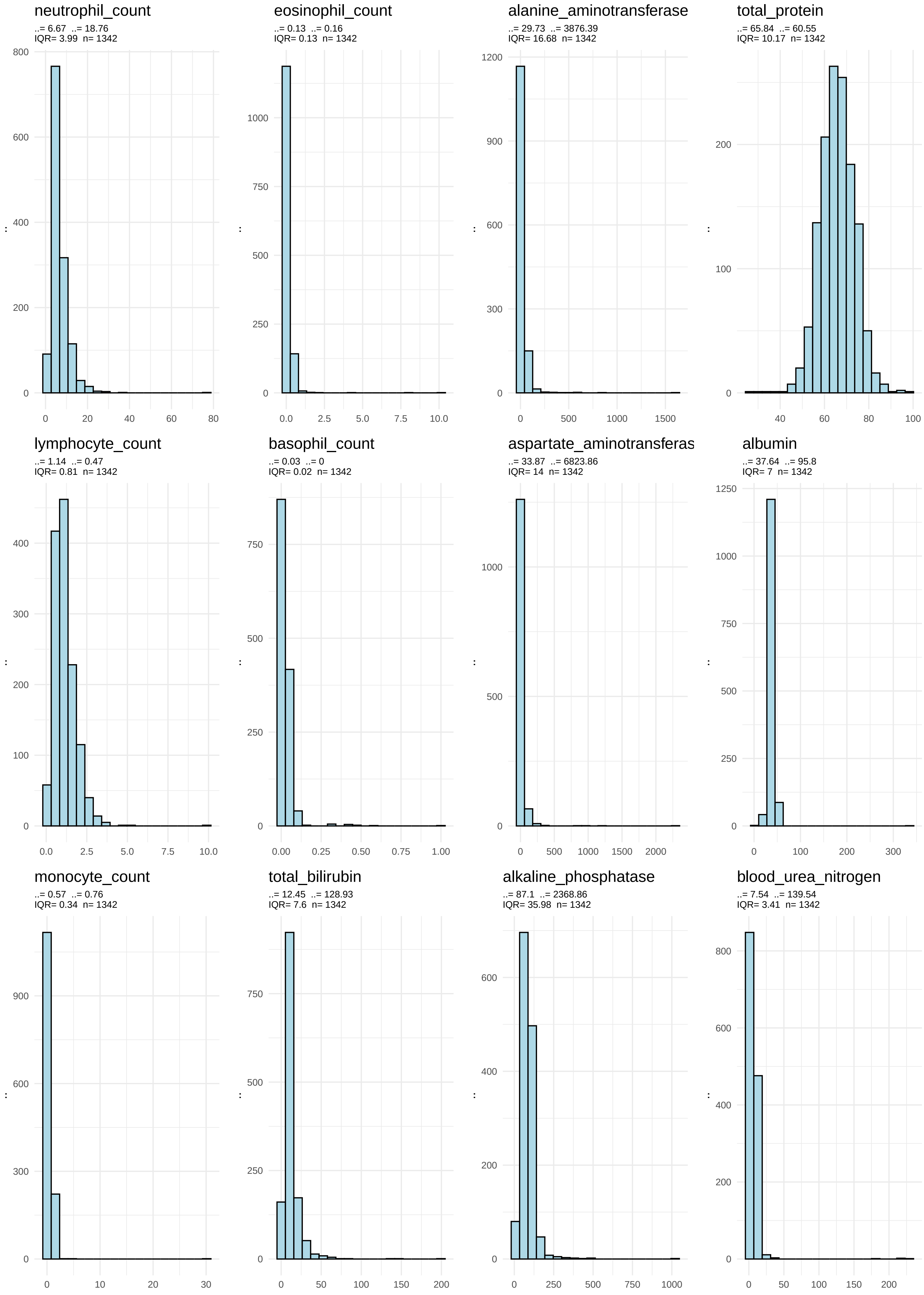
neutrophil_count
..= 6.67 ..= 18.76
IQR= 3.99 n= 1342
eosinophil_count
..= 0.13 ..= 0.16
IQR= 0.13 n= 1342
alanine_aminotransferase
..= 29.73 ..= 3876.39
IQR= 16.68 n= 1342
total_protein
..= 65.84 ..= 60.55
IQR= 10.17 n= 1342
lymphocyte_count
..= 1.14 ..= 0.47
IQR= 0.81 n= 1342
basophil_count
..= 0.03 ..= 0
IQR= 0.02 n= 1342
aspartate_aminotransferas
..= 33.87 ..= 6823.86
IQR= 14 n= 1342
albumin
..= 37.64 ..= 95.8
IQR= 7 n= 1342
monocyte_count
..= 0.57 ..= 0.76
IQR= 0.34 n= 1342
total_bilirubin
..= 12.45 ..= 128.93
IQR= 7.6 n= 1342
alkaline_phosphatase
..= 87.1 ..= 2368.86
IQR= 35.98 n= 1342
blood_urea_nitrogen
..= 7.54 ..= 139.54
IQR= 3.41 n= 1342

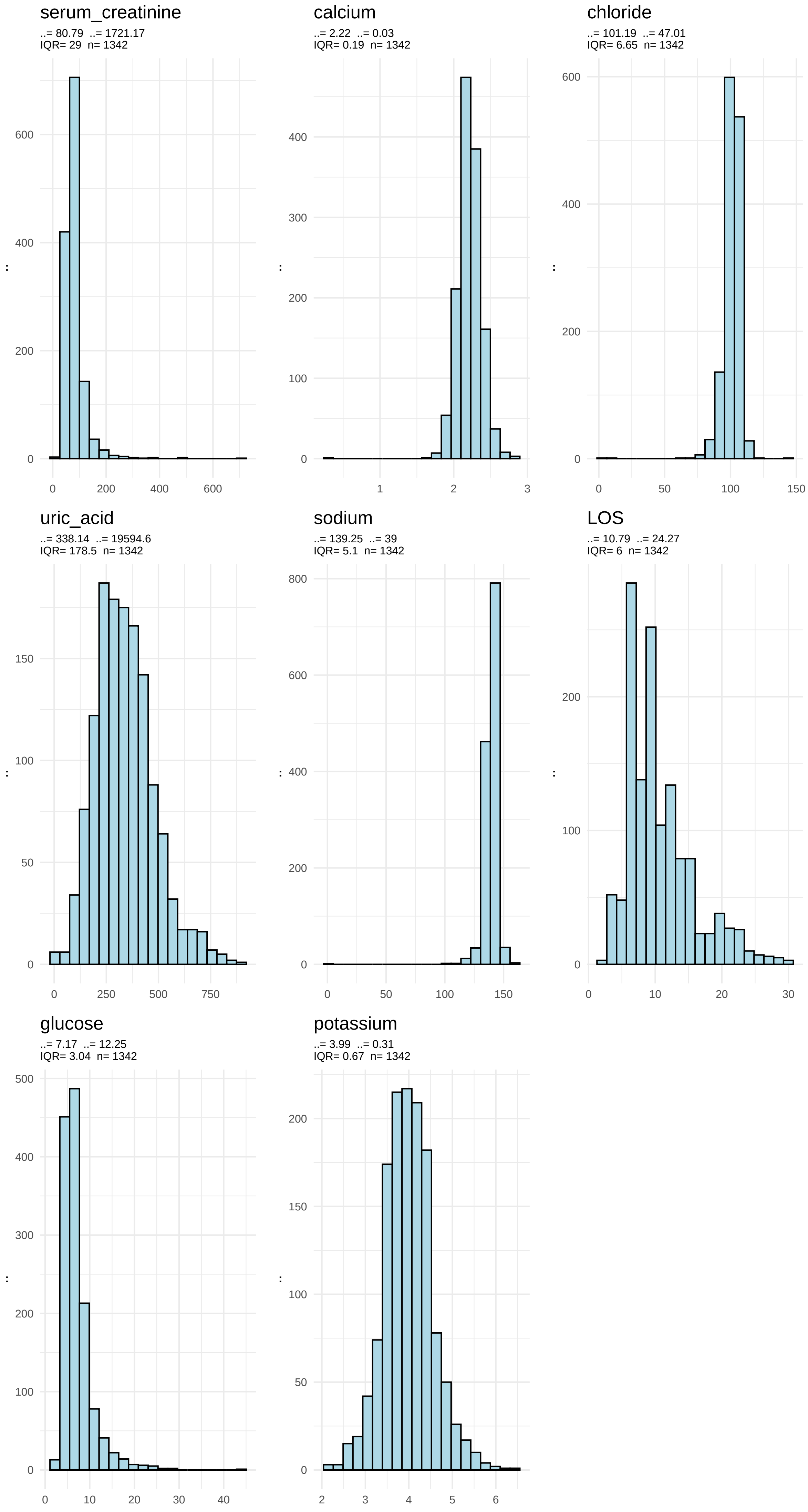
serum_creatinine
..= 80.79 ..= 1721.17
IQR= 29 n= 1342
calcium
..= 2.22 ..= 0.03
IQR= 0.19 n= 1342
chloride
..= 101.19 ..= 47.01
IQR= 6.65 n= 1342
uric_acid
..= 338.14 ..= 19594.6
IQR= 178.5 n= 1342
sodium
..= 139.25 ..= 39
IQR= 5.1 n= 1342
LOS
..= 10.79 ..= 24.27
IQR= 6 n= 1342
glucose
..= 7.17 ..= 12.25
IQR= 3.04 n= 1342
potassium
..= 3.99 ..= 0.31
IQR= 0.67 n= 1342
