# Supplementary material for: Prediction of Prolonged Length of Hospital Stay in Patients with Acute Exacerbations of Chronic Obstructive Pulmonary Disease: An Interpretable Machine Learning Tool
Source: J Clin Med. 2026 Aug 6;15(15):6127. doi: 10.3390/jcm15156127 (PMC13467324; doi:10.3390/jcm15156127)

Supplemental Figure S2. SHAP value plots of every variable in Random Forest

(a-g) SHAP dependence plots of categorical variables: daily inhaled medication use, sputum microbiological examination, antibiotic administration, corticosteroid therapy, diuretic use, ICU admission. (h-i) SHAP dependence plots of continuous variables: oxygenation index, platelet count and neutrophil count.

SHAP: SHapley Additive exPlanations; LOS: length of hospital stay.

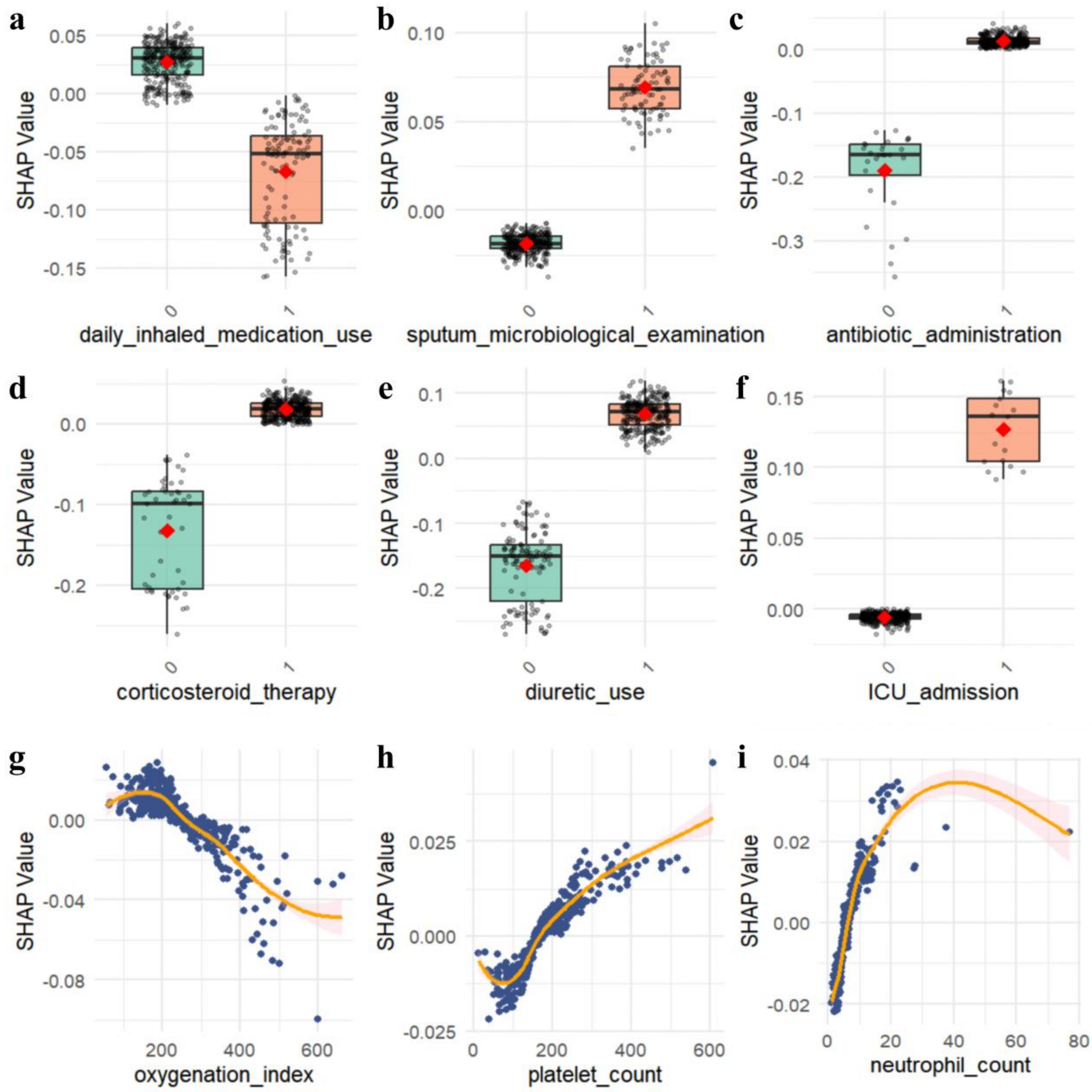

Supplement: Supplementary file 1 [file jcm-15-06127-s001.zip › Supplemental Figure S2 SHAP value plots of every variable in Random Forest.pdf]
