# Supplementary material for: Prediction of Prolonged Length of Hospital Stay in Patients with Acute Exacerbations of Chronic Obstructive Pulmonary Disease: An Interpretable Machine Learning Tool
Source: J Clin Med. 2026 Aug 6;15(15):6127. doi: 10.3390/jcm15156127 (PMC13467324; doi:10.3390/jcm15156127)

Supplemental Figure S3. SHAP summary plot of subgroup analysis

(a) Sub-age (≤65years, ＞65years) analysis. (b) Sub-gender (female, male) analysis. (c) Sub-NT-proBNP (below the maximum detection value, above the maximum detection value) analysis. (d) Sub-eGFR (≥ 60mL/min/1.73m^2, ≤59mL/min/1.73m^2) analysis. (e) Sub-low-risk (age ≤65 years and eGFR0) analysis. SHAP: SHapley Additive exPlanations；NT-proBNP: N-terminal pro-B-type natriuretic peptide. eGFR: estimated glomerular filtration rateand, calculated using the CKD-EPI 2021 creatinine equation; marked as numerical value (≥60mL/min/1.73m^2=0, ≤59mL/min/1.73m^2=1) for statistical analysis.

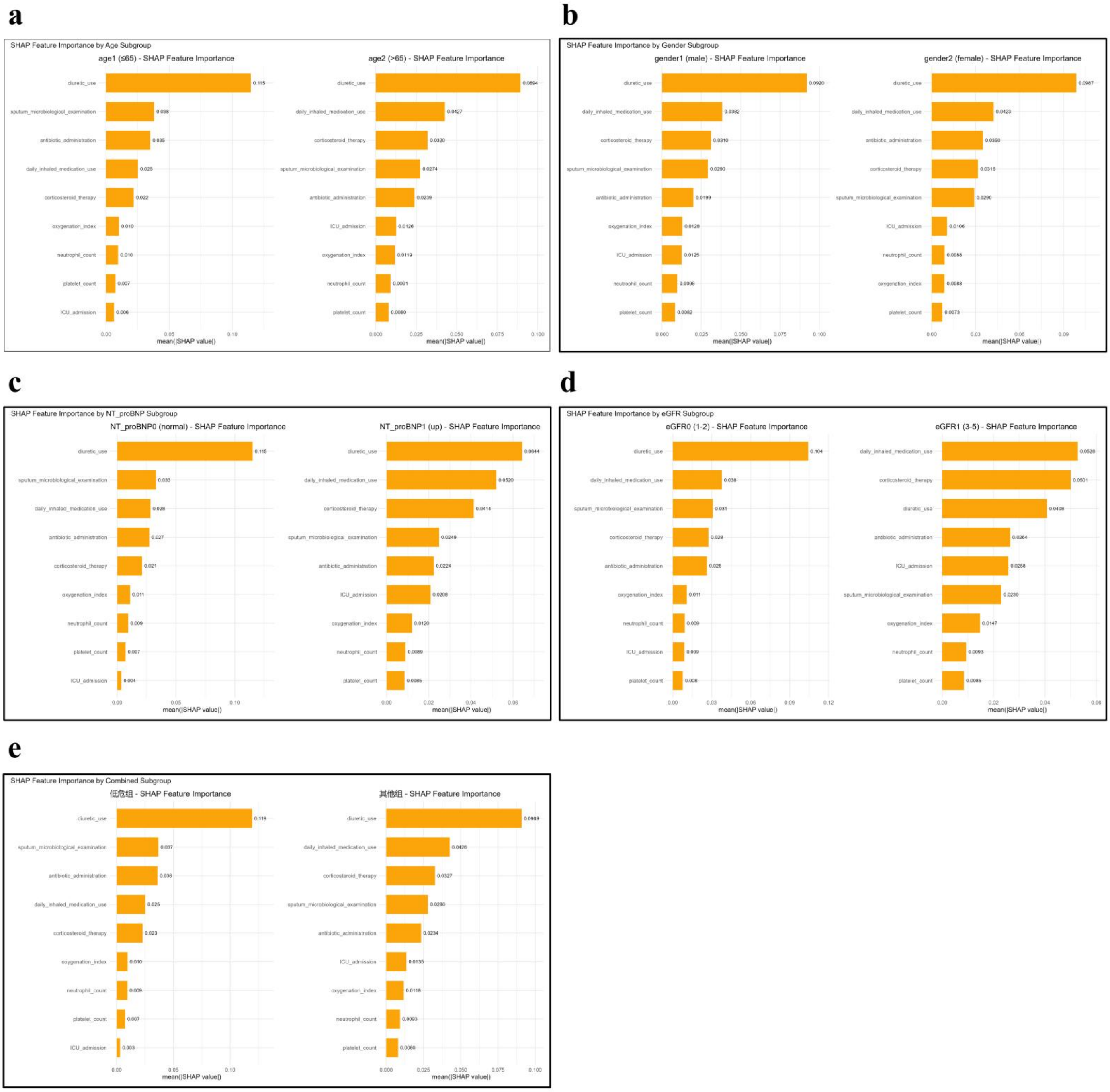

Supplement: Supplementary file 1 [file jcm-15-06127-s001.zip › Supplemental Figure S3. SHAP summary plot of subgroup analysis.pdf]
