# Supplementary material for: Prediction of Prolonged Length of Hospital Stay in Patients with Acute Exacerbations of Chronic Obstructive Pulmonary Disease: An Interpretable Machine Learning Tool
Source: J Clin Med. 2026 Aug 6;15(15):6127. doi: 10.3390/jcm15156127 (PMC13467324; doi:10.3390/jcm15156127)
Supplement: Supplementary file 1 [file jcm-15-06127-s001.zip › Supplemental Figure S4 Sensitivity analyses (LOS7 vs. LOS10).pdf]

Supplemental Figure S4. Sensitivity analyses (LOS7)

(a) ROC curves in test set (LOS7 vs. LOS10). (b) Calibration curves in test set (LOS7 vs. LOS10). (c) DCA plots in test set (LOS7 vs. LOS10). (d) Performance comparison in test set (LOS7 vs. LOS10). (e-g) ROC curves and SHAP summary plot of sub-age (≤65years, ＞65years) analysis in test set. (h-g) ROC curves and SHAP summary plot of sub-gender (female, male) analysis in test set. (k-m) ROC curves and SHAP summary plot of sub-NT-proBNP (below the maximum detection value, above the maximum detection value) analysis in test set. (n-p) ROC curves and SHAP summary plot of sub-eGFR (≥ 60mL/min/1.73m², ≤59mL/min/1.73m²) analysis in test set. (q-s) ROC curves and SHAP summary plot of sub-low-risk (age ≤65 years and eGFR0) analysis in test set.

SHAP: SHapley Additive exPlanations；NT-proBNP: N-terminal pro-B-type natriuretic peptide. eGFR: estimated glomerular filtration rateand, calculated using the CKD-EPI 2021 creatinine equation; marked as numerical value (≥60mL/min/1.73m² =0, ≤59mL/min/1.73m² =1) for statistical analysis.

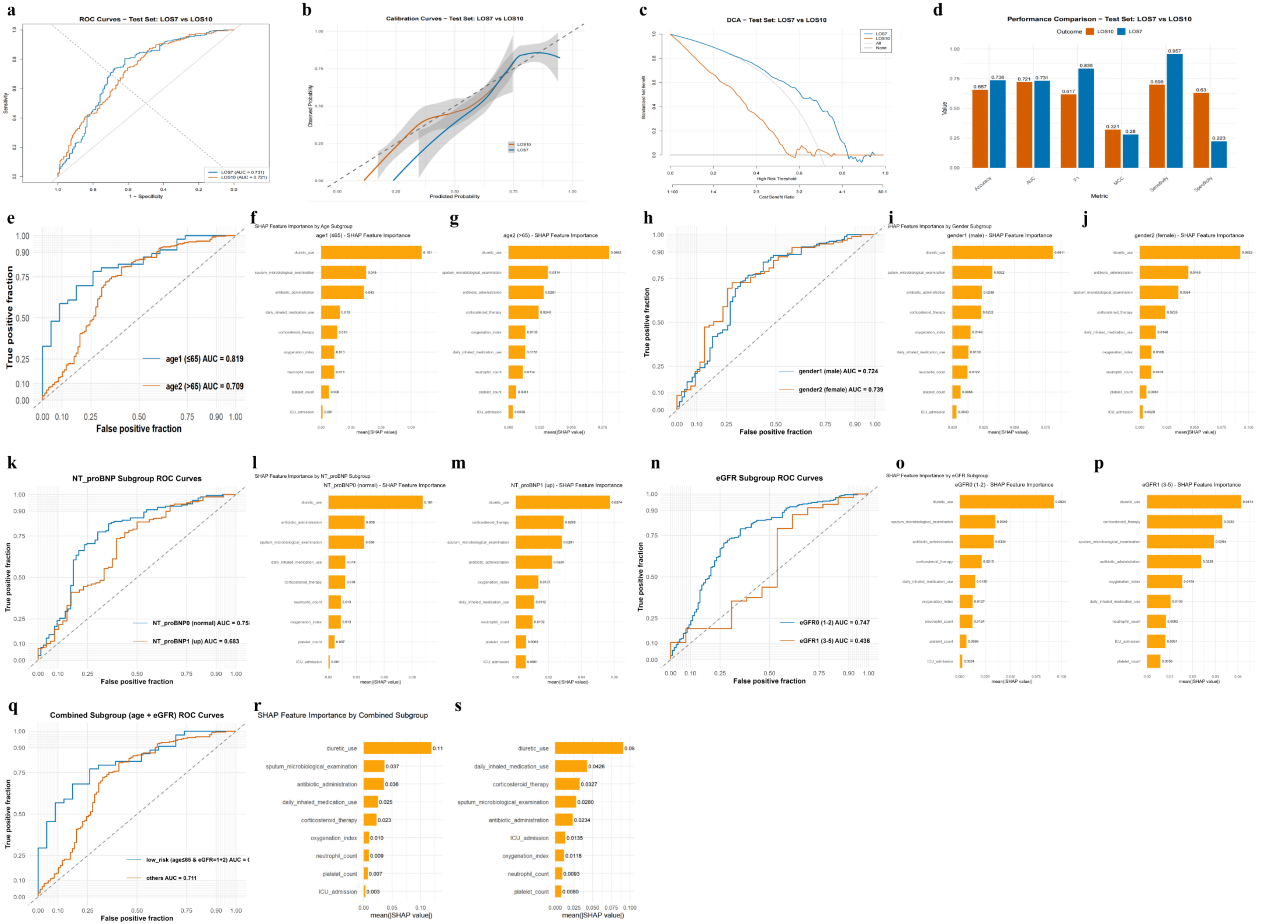
a
ROC Curves – Test Set: LOS7 vs LOS10
Sensitivity
1 – Specificity
LOS7 (AUC = 0.731)
LOS10 (AUC = 0.721)
b
Calibration Curves – Test Set: LOS7 vs LOS10
Observed Probability
Predicted Probability
LOS10
LOS7
c
DCA – Test Set: LOS7 vs LOS10
Standardized Net Benefit
High Risk Threshold
Cost:Benefit Ratio
LOS7
LOS10
All
None
d
Performance Comparison – Test Set: LOS7 vs LOS10
Outcome LOS10 LOS7
Value
Metric
Accuracy
AUC
F1
MCC
Sensitivity
Specificity
0.657 0.736 0.721 0.731 0.617 0.835 0.321 0.28 0.698 0.957 0.63 0.223
e
True positive fraction
False positive fraction
age1 (≤65) AUC = 0.819
age2 (>65) AUC = 0.709
f
SHAP Feature Importance by Age Subgroup
age1 (≤65) - SHAP Feature Importance
g
age2 (>65) - SHAP Feature Importance
h
True positive fraction
False positive fraction
gender1 (male) AUC = 0.724
gender2 (female) AUC = 0.739
i
SHAP Feature Importance by Gender Subgroup
gender1 (male) - SHAP Feature Importance
j
gender2 (female) - SHAP Feature Importance
k
NT_proBNP Subgroup ROC Curves
True positive fraction
False positive fraction
NT_proBNP0 (normal) AUC = 0.75
NT_proBNP1 (up) AUC = 0.683
l
SHAP Feature Importance by NT_proBNP Subgroup
NT_proBNP0 (normal) - SHAP Feature Importance
m
NT_proBNP1 (up) - SHAP Feature Importance
n
eGFR Subgroup ROC Curves
True positive fraction
False positive fraction
eGFR0 (1-2) AUC = 0.747
eGFR1 (3-5) AUC = 0.436
o
SHAP Feature Importance by eGFR Subgroup
eGFR0 (1-2) - SHAP Feature Importance
p
eGFR1 (3-5) - SHAP Feature Importance
q
Combined Subgroup (age + eGFR) ROC Curves
True positive fraction
False positive fraction
low_risk (age≤65 & eGFR=1+2) AUC =
others AUC = 0.711
r
SHAP Feature Importance by Combined Subgroup
diuretic_use 0.11
sputum_microbiological_examination 0.037
antibiotic_administration 0.036
daily_inhaled_medication_use 0.025
corticosteroid_therapy 0.023
oxygenation_index 0.010
neutrophil_count 0.009
platelet_count 0.007
ICU_admission 0.003
mean(|SHAP value|)
s
diuretic_use 0.09
daily_inhaled_medication_use 0.0426
corticosteroid_therapy 0.0327
sputum_microbiological_examination 0.0280
antibiotic_administration 0.0234
ICU_admission 0.0135
oxygenation_index 0.0118
neutrophil_count 0.0093
platelet_count 0.0080
mean(|SHAP value|)
