# Supplementary material for: Prediction of Prolonged Length of Hospital Stay in Patients with Acute Exacerbations of Chronic Obstructive Pulmonary Disease: An Interpretable Machine Learning Tool
Source: J Clin Med. 2026 Aug 6;15(15):6127. doi: 10.3390/jcm15156127 (PMC13467324; doi:10.3390/jcm15156127)
Supplement: Supplementary file 1 [file jcm-15-06127-s001.zip › Supplemental Table S1 Baseline characteristics of three hospitals.pdf]

Supplemental Table S1. Baseline characteristics of three hospitals

variables	total (n=1342)	West China Hospital, Sichuan University （n=657）	Luojiang District People's Hospital （n=401）	People's Hospital of Leshan （n=284）
gender				
male=1	915(68.2%)	427(65.0%)	284(70.8%)	204(71.8%)
female=2	427(31.8%)	230(35.0%)	117(29.2%)	80(28.2%)
age (years)	73.55±10.00	73.98±9.49	73.34±9.83	72.84±11.29
marital status				
married = 1	1199(89.3%)	564(85.8%)	371(92.5%)	264(93.0%)
other(unmarried/divorced/widowed)=2	143(10.7%)	93(14.2%)	30(7.5%)	20(7.0%)
family history of COPD				
no=0	1335(99.5%)	654(99.5%)	399(99.5%)	282(99.3%)
yes=1	7(0.5%)	3(0.5%)	2(0.5%)	2(0.7%)
smoking history				
non-smoker=0	117(8.7%)	41(6.2%)	33(8.2%)	43(15.1%)
ex-smoker/current smoker=1	1225(91.3%)	616(93.8%)	368(91.8%)	241(84.9%)
daily inhaled medication use				
no=0	982(73.2%)	427(65.0%)	302(75.3%)	253(89.1%)
yes=1	360(26.8%)	230(35.0%)	99(24.7%)	31(10.9%)
heart rate (bmp/min)	91.39±18.64	90.86±17.55	92.46±19.68	91.08±19.57
respiratory rate (bmp/min)	20[20,21]	20[20,21]	20[20.21]	20[20.21]
diastolic blood pressure (mmHg)	77[68,85]	77[69, 86]	77[69, 85]	75[66, 83]
systolic blood pressure (mmHg)	128[113,142]	127[114, 141]	129[113,143]	128[113,142]
potential of Hydrogen	7.41[7.38,7.44]	7.41[7.38, 7.44]	7.41[7.38, 7.44]	7.42[7.38, 7.45]
oxygenation index	255.01±94.77	261.82±95.77	247.14±92.09	250.38±95.39
arterial carbon dioxide tension (mmHg)	43.00[37.00,51.85]	43.00[37.60, 51.70]	42.40[36.80,51.00]	43.00[36.45,54.38]
bicarbonate ion (mmol/L)	28.23±6.43	28.12±6.15	27.99±6.18	28.79±7.45
hemoglobin concentration (g/L)	134.00[119.00,147.00]	135.00[119.00, 146.00]	135.00[121.00,148.00]	133.00[115.00,148.00]
platelet count (10^9/L)	171.00[128.00,222.00]	162.00[120.00, 212.00]	174.00[134.00,230.00]	177.00[136.00,237.00]
white blood cell count (10^9 /L)	7.59[5.75,9.89]	7.24[5.54, 9.53]	7.65[6.01,10.19]	8.00[6.04,10.75]
neutrophil count (10^9 /L)	5.64[4.02,8.02]	5.42[3.82, 7.41]	5.81[4.27,7.98]	6.08[4.45,9.06]
lymphocyte count (10^9 /L)	1.02[0.66,1.47]	1.06[0.69, 1.49]	1.02[0.68,1.51]	0.93[0.60,1.33]
monocyte count (10^9 /L)	0.49[0.34,0.68]	0.49[0.35, 0.66]	0.50[0.35,0.71]	0.49[0.32,0.69]
eosinophil count (10^9 /L)	0.06[0.02,0.15]	0.06[0.02, 0.16]	0.06[0.02,0.14]	0.06[0.01,0.15]
basophil count (10^9 /L)	0.02[0.01,0.03]	0.02[0.01, 0.03]	0.02[0.01,0.03]	0.02[0.01,0.03]
total bilirubin (umol/L)	9.70[6.80,14.40]	9.70[6.70, 13.80]	9.60[7.00,14.80]	10.30[6.70,15.10]
alanine aminotransferase (U/L)	19.00[13.00,29.72]	18.88[13.55,28.91]	20.00[13.00,29.82]	19.00[12.00,32.00]
aspartate aminotransferase (U/L)	23.00[18.00,32.00]	24.00[19.00, 32.00]	23.00[18.00,32.00]	21.00[17.00,29.00]
alkaline phosphatase (U/L)	82.00[65.00,101.00]	85.33[69.00, 104.63]	79.00[61.00, 98.00]	78.00[61.00, 101.00]
total protein (g/L)	65.84±7.78	66.85±7.8057	65.34±7.86	64.20±7.84
albumin (g/L)	37.70[34.00,41.00]	38.20[34.80,41.70]	37.60[34.20,40.60]	36.15[32.18,40.10]
blood urea nitrogen (mmol/L)	6.20[4.70,8.11]	6.10[4.73,8.05]	6.20[4.61,8.12]	6.40[4.81,8.15]
serum creatinine (umol/L)	72.80[60.00,89.00]	73.00[61.00, 88.10]	72.00[61.00, 91.00]	72.00[58.00, 89.00]
uric acid (umol/L)	338.14±139.98	346.79±128.35	338.36±142.57	317.84±159.20
calcium (mmol/L)	2.21[2.12,2.31]	2.20[2.12,2.31]	2.22[2.18,2.23]	2.21[2.11,2.32]

sodium (mmol/L)	140.00[137.00,142.20]	140.00[137.00,142.40]	140.00[137.00,142.00]	140.00[137.00,142.00]
potassium (mmol/L)	3.99±0.56	3.96±0.56	3.99±0.53	4.05±0.55
chloride (mmol/L)	102.00[98.30,105.00]	102.30[99.20,105.30]	101.60[97.40,105.00]	101.00[97.80,104.00]
glucose (mmol/L)	6.27[5.05,8.10]	6.23[5.06,8.00]	6.23[4.96,8.07]	6.34[5.17,8.30]
NT-proBNP (pg/mL)				
below the maximum detection value =0	735 (51.77%)	369 (56.2%)	192 (47.9%)	174 (61.3%)
above the maximum detection value =1	607 (45.23%)	288(43.8%)	209 (52.1%)	110 (38.7%)
eGFR (mL/min/1.73m²)				
G1-G2(⩾60)=0	1171 (87.3%)	572 (87.1%)	353 (88.1%)	353 (88.1%)
G3-G5 (⩽59)=1	171 (12.7%)	85 (12.9%)	48 (11.9%)	48 (11.9%)
sputum microbiological examination				
negative =0	1061(79.1%)	558(84.9%)	315(78.5%)	188(66.2%)
positive=1	281(20.9%)	99(15.1%)	86(21.5%)	96(33.8%)
antibiotic administration				
no=0	97(7.2%)	86(13.1%)	10(2.5%)	1(0.4%)
yes=1	1245(92.8%)	571(86.9%)	391(97.5%)	283(99.6%)
corticosteroid therapy				
no=0	173(12.9%)	128(19.5%)	36(9.0%)	9(3.2%)
yes=1	1169(87.1%)	529(80.5%)	365(91.0%)	275(96.8%)
diuretic use				
no=0	424(31.6%)	308(46.9%)	94(23.4%)	22(7.8%)
yes=1	918(68.4%)	349(53.1%)	307(76.6%)	262(92.2%)
non-invasive ventilation application				
no=0	1084(80.8%)	88(13.3%)	325(81.1%)	190(67.0%)
yes=1	258(19.2%)	569(88.6%)	76(18.9%)	94(33.0%)
ICU admission				
no=0	1285(95.8%)	645(98.1%)	386(96.1%)	255(89.7%)
yes=1	57(4.2%)	12(1.8%)	16(3.9%)	29(27.2%)
LOS (days)	9[7, 13]	7[6, 8]	11[10,13]	18[15,21]

Values are expressed as the mean ± standard deviation or the median [interquartile range]. LOS: length of hospital stay.
