# Supplementary material for: Prediction of Prolonged Length of Hospital Stay in Patients with Acute Exacerbations of Chronic Obstructive Pulmonary Disease: An Interpretable Machine Learning Tool
Source: J Clin Med. 2026 Aug 6;15(15):6127. doi: 10.3390/jcm15156127 (PMC13467324; doi:10.3390/jcm15156127)
Supplement: Supplementary file 1 [file jcm-15-06127-s001.zip › Supplemental Table S2 Baseline characteristics of training set.pdf]

Supplemental Table S2. Baseline characteristics of training set

variables	training set (n=940)	LOS⩽10 (n=535)	LOS＞10 (n=405)
gender			
male=1	651(69.3%)	355(66.4%)	296(73.1%)
female=2	289(30.7%)	180(33.6%)	109(26.9%)
age (years)	73.48±9.89	73.93±9.40	72.88±10.49
marital status			
married = 1	840(89.4%)	460(86.0%)	380(93.8%)
other(unmarried/divorced/widowed)=2	100(10.6%)	75(14.0%)	25(6.2%)
family history of COPD			
no=0	936(99.6%)	532(99.4%)	404(99.8%)
yes=1	4(0.4%)	3(0.56%)	1(0.2%)
smoking history			
non-smoker=0	80(8.5%)	33(6.2%)	47(11.6%)
ex-smoker/current smoker=1	860(91.5%)	502(93.8%)	358(88.4%)
daily inhaled medication use			
no=0	696(74.0%)	357(66.7%)	339(83.7%)
yes=1	244(26.0%)	178(33.2%)	66(16.3%)
heart rate (bmp/min)	91.84±18.18	91.21±17.53	92.68±18.99
respiratory rate (bmp/min)	20[20,21]	20[20,21]	20[20,21]
diastolic blood pressure (mmHg)	77[68, 80]	76[69, 85]	77[68,84]
systolic blood pressure (mmHg)	128[113, 142]	128[113, 141]	128[113,143]
potential of Hydrogen	7.41[7.38, 7.44]	7.41[7.38, 7.44]	7.41[7.38,7.45]
oxygenation index	255.37±93.71	263.76±96.68	244.30±88.55
arterial carbon dioxide tension (mmHg)	42.80[37.00, 52.00]	43.00[37.40, 52.00]	42.40[36.50,51.60]
bicarbonate ion (mmol/L)	28.19±6.54	28.26±6.39	28.10±6.37
hemoglobin concentration (g/L)	135.00[119.00, 147.00]	134.00[120.00, 145.00]	135.00[119.00,150.00]
platelet count (10^9/L)	170.00[129.00, 223.75]	161.00[120.00, 211.00]	179.00[140.00,243.00]
white blood cell count (10^9 /L)	7.41[5.71, 9.81]	7.14[5.36,9.34]	7.79[6.03,10.42]
neutrophil count (10^9 /L)	5.52[3.99, 7.89]	5.28[3.81,7.25]	5.92[4.25,8.59]
lymphocyte count (10^9 /L)	1.03[0.68, 1.48]	1.06[0.72,1.52]	0.99[0.63,1.45]
monocyte count (10^9 /L)	0.50[0.34, 0.68]	0.50[0.35,0.67]	0.49[0.35,0.70]
eosinophil count (10^9 /L)	0.06[0.02, 0.15]	0.06[0.02,0.16]	0.06[0.01,0.15]
basophil count (10^9 /L)	0.02[0.01, 0.03]	0.02[0.01, 0.03]	0.02[0.01, 0.03]
total bilirubin (umol/L)	9.70[6.80, 14.68]	9.80[6.80,14.50]	9.70[6.70,14.80]
alanine aminotransferase (U/L)	19.00[13.00,29.23]	18.88[13.00, 29.00]	19.00[12.99,29.81]
aspartate aminotransferase (U/L)	23.00[18.00, 31.75]	23.00[18.00,32.00]	22.00[17.00,30.00]
alkaline phosphatase (U/L)	81.00[65.00, 99.63]	84.16[68.00,101.00]	78.00[61.00,99.00]
total protein (g/L)	65.89±7.80	66.74±7.57	64.78±7.96
albumin (g/L)	37.70[34.00,40.80]	38.30[35.10, 41.60]	36.70[33.10,39.90]
blood urea nitrogen (mmol/L)	6.19[4.69,8.00]	6.07[4.69, 8.00]	6.23[4.70,7.92]
serum creatinine (umol/L)	72.80[60.00, 88.98]	73.00[60.00, 88.20]	72.60[60.00,89.40]

uric acid (umol/L)	338.44±139.94	345.00±132.34	329.78±149.12
calcium (mmol/L)	2.21[2.12,2.31]	2.20[2.12, 2.30]	2.23[2.12,2.32]
sodium (mmol/L)	140.00[137.00,142.18]	139.30[140.00, 142.30]	140.00[137.00,142.00]
potassium (mmol/L)	3.98±0.57	3.97±0.59	4.01±0.55
chloride (mmol/L)	102.00[98.73,105.00]	102.30[99.20,105.15]	101.20[97.70,104.90]
glucose (mmol/L)	6.20[4.99,8.13]	6.22[4.98, 8.14]	6.20[5.05,8.08]
NT-proBNP (pg/mL)			
below the maximum detection value =0	520 (55.32%)	301 (56.3%)	219 (54.1%)
above the maximum detection value =1	420 (44.68%)	234 (43.7%)	186 (45.1%)
eGFR (mL/min/1.73m^2)			
G1-G2(≥60)=0	830 (88.30%)	475 (88.8%)	485 (87.7%)
G3-G5 (≤59)=1	110 (11.70%)	60 (11.2%)	50 (12.3%)
sputum microbiological examination			
negative =0	744(79.1%)	451(84.3%)	293(72.4%)
positive=1	196(20.9%)	84(15.7%)	112(27.6%)
antibiotic administration			
no=0	69(7.3%)	64(11.9%)	5(1.2%)
yes=1	871(92.7%)	471(88.1%)	400(98.8%)
corticosteroid therapy			
no=0	125(13.3%)	99(18.5%)	26(6.4%)
yes=1	815(86.7%)	436(91.5%)	379(93.6%)
diuretic use			
no=0	307(32.7%)	247(46.2%)	60(14.8%)
yes=1	633(67.3%)	288(53.8%)	345(85.2%)
non-invasive ventilation application			
no=0	758(80.6%)	458(85.6%)	300(74.1%)
yes=1	182(19.4%)	77(14.4%)	105(25.9%)
ICU admission			
no=0	901(95.9%)	525(98.1%)	376(92.8%)
yes=1	39(4.1%)	10(1.9%)	29(7.2%)
LOS (days)	10[7,14]	7[8, 9]	14[12,18]

Values are expressed as the mean ± standard deviation or the median [interquartile range]. LOS: length of hospital stay.
