# Supplementary material for: Prediction of Prolonged Length of Hospital Stay in Patients with Acute Exacerbations of Chronic Obstructive Pulmonary Disease: An Interpretable Machine Learning Tool
Source: J Clin Med. 2026 Aug 6;15(15):6127. doi: 10.3390/jcm15156127 (PMC13467324; doi:10.3390/jcm15156127)
Supplement: Supplementary file 1 [file jcm-15-06127-s001.zip › Supplemental Table S3 Sensitivity analyses (LOS7 vs. LOS10).pdf]

Supplemental Table S3. Sensitivity analyses (LOS7 vs. LOS10)

Outcome	Dataset/ Subgroup	AUC	AUC_95CI_L	AUC_95CI_H	Accuracy	Balanced Accuracy	Sensitivity	Specificity	PPV	NPV	F1	MCC	Brier	Kappa
LOS7	Train	0.802	0.773	0.832	0.771	0.635	0.951	0.318	0.779	0.72	0.856	0.367	0.159	0.324
LOS7	Test	0.731	0.673	0.788	0.736	0.59	0.957	0.223	0.741	0.692	0.835	0.28	0.176	0.224
LOS10	Train	0.778	0.748	0.807	0.709	0.715	0.76	0.669	0.635	0.787	0.692	0.426	0.194	0.42
LOS10	Test	0.721	0.672	0.771	0.657	0.664	0.698	0.63	0.552	0.761	0.617	0.321	0.206	0.313
LOS7	age1 (≤65)	0.819	0.718	0.919	0.725	0.713	0.935	0.304	0.729	0.7	0.819	0.32	0.17	0.278
LOS7	age2 (>65)	0.709	0.642	0.776	0.739	0.728	0.962	0.204	0.743	0.69	0.839	0.268	0.178	0.209
LOS10	age1 (≤65)	0.794	0.683	0.906	0.71	0.701	0.75	0.683	0.618	0.8	0.677	0.425	0.182	0.419
LOS10	age2 (>65)	0.703	0.648	0.758	0.646	0.634	0.687	0.619	0.539	0.753	0.604	0.299	0.212	0.292
LOS7	gender1 (male)	0.724	0.652	0.797	0.735	0.711	0.962	0.192	0.74	0.682	0.836	0.255	0.175	0.195
LOS7	gender2 (female)	0.739	0.644	0.833	0.739	0.719	0.947	0.279	0.744	0.706	0.833	0.319	0.179	0.271
LOS10	gender1 (male)	0.73	0.671	0.789	0.655	0.648	0.727	0.604	0.567	0.756	0.637	0.327	0.205	0.318
LOS10	gender2 (female)	0.695	0.601	0.788	0.659	0.642	0.633	0.674	0.517	0.769	0.569	0.296	0.21	0.292
LOS7	NT_proBNP0 (normal)	0.758	0.685	0.831	0.707	0.712	0.93	0.274	0.714	0.667	0.807	0.278	0.182	0.238
LOS7	NT_proBNP1 (up)	0.683	0.59	0.776	0.77	0.849	0.986	0.146	0.77	0.778	0.864	0.268	0.169	0.179
LOS10	NT_proBNP0 (normal)	0.735	0.667	0.803	0.698	0.704	0.753	0.664	0.575	0.817	0.652	0.404	0.198	0.393
LOS10	NT_proBNP1 (up)	0.697	0.623	0.772	0.61	0.632	0.641	0.587	0.526	0.696	0.578	0.225	0.216	0.221
LOS7	eGFR0 (1-2)	0.747	0.688	0.807	0.724	0.714	0.953	0.231	0.728	0.694	0.825	0.279	0.179	0.224
LOS7	eGFR1 (3-5)	0.436	0.232	0.64	0.803	0.794	0.979	0.154	0.81	0.667	0.887	0.252	0.161	0.185

Outcome	Dataset/ Subgroup	AUC	AUC_95CI_L	AUC_95CI_H	Accuracy	Balanced Accuracy	Sensitivity	Specificity	PPV	NPV	F1	MCC	Brier	Kappa
LOS10	eGFR0 (1-2)	0.738	0.685	0.791	0.674	0.669	0.72	0.646	0.562	0.785	0.631	0.356	0.201	0.348
LOS10	eGFR1 (3-5)	0.602	0.457	0.748	0.557	0.561	0.593	0.529	0.5	0.621	0.542	0.121	0.237	0.12
LOS7	low risk (age≤65 & eGFR=1+2)	0.81	0.706	0.915	0.716	0.712	0.932	0.304	0.719	0.7	0.812	0.315	0.175	0.273
LOS7	others	0.711	0.644	0.778	0.74	0.767	0.962	0.204	0.745	0.69	0.84	0.269	0.177	0.209
LOS10	low risk (age≤65 & eGFR=1+2)	0.779	0.661	0.896	0.701	0.717	0.731	0.683	0.594	0.8	0.655	0.404	0.184	0.397
LOS10	others	0.707	0.653	0.762	0.648	0.641	0.692	0.619	0.544	0.753	0.609	0.304	0.211	0.297

NT-proBNP: N-terminal pro-B-type natriuretic peptide. eGFR: estimated glomerular filtration rateand, calculated using the CKD-EPI 2021 creatinine equation; marked as numerical value (≥60mL/min/1.73m²=0, ≤59mL/min/1.73m²=1) for statistical analysis..
